# Associations between self-rated health, sickness behaviour and inflammatory markers in primary care patients with allergic asthma: a longitudinal study

**DOI:** 10.1038/s41533-017-0068-0

**Published:** 2017-12-18

**Authors:** Karin Lodin, Mats Lekander, Jörgen Syk, Kjell Alving, Anna Andreasson

**Affiliations:** 1 Department of Neurobiology, Care Sciences and Society, Karolinska Institute, Stockholm, Sweden; 2 Department of Clinical Neuroscience, Karolinska Institute, Stockholm, Sweden; 3 Stress Research Institute, Stockholm University, Stockholm, Sweden; 4 Centre for Allergy Research, Karolinska Institute, Stockholm, Sweden; 5 Academic Primary Health Care Centre, Stockholm, Sweden; 6 Department of Women’s and Children’s Health, Uppsala University, Uppsala, Sweden; 7 Department of Psychology, Macquarie University, North Ryde, Australia

## Abstract

Allergic asthma is a chronic inflammatory disorder associated with elevated levels of immunoglobulin E (IgE), serum eosinophilic cationic protein (S-ECP), plasma eosinophil-derived neurotoxin (P-EDN) and fraction of exhaled nitric oxide (F_E_NO). Poor self-rated health and sickness behaviour has repeatedly been associated with inflammatory markers, but the nature of this relationship in chronic inflammatory disease is not known. Likewise, such findings largely rely on cross-sectional investigations. Self-rated health (How would you rate your general state of health?), sickness behaviour (mean rating of satisfaction with energy, sleep, fitness, appetite and memory), IgE, S-ECP, P-EDN, and F_E_NO were assessed in 181 non-smoking primary care patients with asthma in a 1-year longitudinal study. Associations between repeated measurements were calculated using mixed regression models and Spearman’s correlations for change scores. Poor self-rated health was associated with high levels of seasonal IgE (*p* = 0.05) and food IgE (*p* = 0.04), but not total IgE or inflammatory markers. An increase over 1 year in perennial IgE was associated with a worsening of self-rated health (*ρ* = 0.16, *p* = 0.04). Poor self-rated health was associated with more pronounced sickness behaviour (*p* < 0.001), and a worsening in sickness behaviour was associated with a worsening of self-rated health over time (*ρ* = 0.21, *p* = 0.007). The study corroborates the importance of sickness behaviour as a determinant of self-rated health by showing that these factors co-vary over a 1-year period in a group of patients with allergic asthma. The importance of specific IgE for perceived health in primary care patients with mild to moderate asthma needs further investigation.

## Allergic asthma: association between perceived health and inflammation

Patient perceptions of their overall health are associated with increased sickness behaviours and spikes in specific allergy antibody levels. Allergic asthma is a chronic inflammatory condition that can trigger associated “sickness behaviours” including extreme fatigue, increased pain sensitivity and anorexia. Certain inflammatory markers are increased in patients with poor self-rated health, but the relationship between perceived health and inflammation is unclear. Karin Lodin at the Karolinska Institute, Sweden, and co-workers investigated levels of four key inflammatory markers alongside self-rated health and sickness behaviours in 181 allergic asthma patients over 12 months. Poor self-rated health was associated with more pronounced sickness behaviours and with high levels of specific immunoglobin E antibodies, particularly those related to food and seasonal changes. However, there was no association between poor self-rated health and the three other pro-inflammatory markers.

## Introduction

Health care use of patient-reported outcomes, directly reported by the patient without interpretation of the response by a clinician or anyone else,^1^ has been related to increased cost-effectiveness and improved survival.^1,2^ In asthma, patient-reported outcomes have potential in assessing the impact of the disease and its treatment on health-related outcomes from the perspective of the patient.^3^ Therefore, a better understanding of determinants of central patient-reported outcomes in asthma, such as ratings of overall health, is warranted.

Self-rated health refers to how individuals evaluate their general health status through a single item question. In spite of its brevity, it provides additional information to that obtained from other sources, including medical examinations. In fact, self-rated health equals and in many cases surpasses objective measures in predicting objective long-term health outcomes such as mortality and morbidity.^4^ Poor self-rated health has been associated with higher levels of inflammatory markers, including erythrocyte sedimentation rate,^5^ C-reactive protein^6^ and the innate pro-inflammatory cytokines IL-6 and TNF-alpha.^7–9^ However, the relation between health estimates and the allergic inflammation is unclear.

Allergic asthma is a chronic inflammatory disorder of the airways with both systemic inflammation with increased levels of IgE and local eosinophilic inflammation with the release of eosinophilic granular proteins such as eosinophil-derived neurotoxin (EDN) and eosinophil cationic protein (ECP).^10,11^ Elevated levels of EDN and ECP reflect eosinophilic activity in the lungs as well as severity and activity of asthma.^12^ The local eosinophilic inflammation in the airways is also reflected in the fraction of exhaled nitric oxide (F_E_NO), which is increased in patients with allergic asthma.^13,14^

Innate pro-inflammatory cytokines are central in orchestrating a set of acute behavioural changes, referred to as sickness behaviour.^15^ If persisting, inflammation is believed to contribute to ill-health^16^ and poor perceived health.^8,9,17,18^ Sickness behaviour includes symptoms such as fatigue, malaise, increased pain sensitivity, anergy, anorexia, fever and anhedonia,^19^ resembling symptoms that correlate with poor self-rated health.^8,20^ Thus, inflammation and associated sickness behaviour have been suggested important determinants for self-rated health.^8,21^

We have previously reported that persons with a diagnosis of asthma report worse self-rated health than persons without asthma.^22^ In addition, patients with asthma often suffer from co-morbid problems such as fatigue^23^ or psychiatric symptoms.^19^ However, it has not yet been studied if either local or systemic allergic inflammation in chronic asthma is associated with poor self-rated health and sickness behaviour in a similar way as have been shown for innate pro-inflammatory markers.^8,9,20^ In addition, most previous studies have been cross-sectional, meaning that little is known about co-variation over time between inflammation and subjective health outcomes.

Here, we investigated sickness behaviour and asthma-specific inflammatory markers, i.e., FENO, IgE, S-ECP and P-EDN, as determinants for self-rated health in primary care patients with chronic allergic asthma followed over 12 months. We hypothesized that both local and systemic inflammatory markers as well as higher ratings of sickness behaviour would be related to poor self-rated health. In addition, we hypothesized that changes in levels of inflammatory markers and sickness behaviour would be mirrored in corresponding changes in self-rated health.

## Results

### Study group characteristics and changes between baseline and follow-up

Study group characteristics at baseline are presented in Table 1. Overall, the participants reported rather good health without pronounced sickness behaviour at the start of the study (Table 1). Self-rated health (*b*: −0.09, 95% CI −0.21; 0.03, *p* = 0.13), F_E_NO (*b*: −0.12, 95% CI −0.32; 0.08, *p* = 0.26), S-ECP (*b*: −5.43, 95% CI −11.70; 0.84, *p* = 0.09) and P-EDN (*b*: 0.28, 95% CI −1.23; 1.79, *p* = 0.72) did not change significantly from baseline to follow-up. However, there was a significant reduction in sickness behaviour (*b*: −0.10, 95% CI −0.20; −0.00, *p* = 0.04) and all groups of IgE decreased significantly over the course of the study, including perennial IgE (*b*: −6.40, 95% CI −9.26; −3.54, *p* < 0.001), seasonal IgE (*b*: −2.31, 95% CI −3.41; −1.22, *p* < 0.001), food IgE (*b*: −1.45, 95% CI −2.67; −0.23, *p* = 0.02) and total IgE (*b*: −26.32, 95% CI −44.59; −8.05; *p* < 0.005).

**Table 1:** Demographic factors, self-rated health, sickness behaviour and inflammatory markers in men and women at baseline

	Women				Men			
	*n*	Range	Mean	SD	*n*	Range	Mean	SD
Age	87	19–63	41.4	11.9	94	18–64	40.6	12.8
BMI	83	17.6–45.2	26.7	6.0	93	18.7–39.4	26.5	3.8
Education	82	1–3	2.4	0.7	89	1–3	2.4	0.7
Self-rated health	86	1–5	2.1	0.8	93	1–4	2.0	0.8
Sickness behaviour^a^	85	1–5.8	3.1	1.0	93	1.2–5.2	3.0	0.9
F_E_NO (ppb)^b^	85	0.4–8.1	1.8	1.5	89	0.3–13.6	1.7	1.7
Perennial IgE (kU/l)	76	0.1–478.1	41.4	76.2	82	0.1–182.2	24.4	31.9
Seasonal IgE (kU/l)	76	0.0–107.9	13.5	20.9	82	0.0–180.3	18.1	34.1
Food IgE (kU/l)	76	0.0–173.2	11.3	26.9	82	0.1–105.0	10.5	21.0
Total IgE (kU/l)	76	0.2–504.2	66.1	99.9	82	0.2–411.7	52.9	71.0
S-ECP	76	2.0–71.4	16.2	12.1	82	2.1–70.8	15.8	13.9
P-EDN	76	6.3–216.5	26.0	36.4	82	9.4–745.6	50.7	120.1

*n* number of individual patients, *BMI* body mass index, *F*_*E*_ *NO* exhaled fraction of nitric oxide, *S-ECP* serum eosinophil cationic protein, *P-EDN* plasma eosinophil-derived neurotoxin

^a^ Composite variable of rating of energy, sleep, memory, fitness and appetite

^b^ F_E_NO—values are reported as mean value from two successive measurements divided by predicted normal F_E_NO values in non-atopic adult subjects adjusted for height and age

### Association between self-rated health, inflammatory factors and sickness behaviour

Poor self-rated health was significantly associated with higher levels of seasonal IgE and higher levels of food IgE in both men and women, independent of age and BMI, but not with perennial IgE or total IgE levels (Table 2). There was no significant association between self-rated health and F_E_NO, S-ECP or P-EDN. The association between higher levels of food and seasonal IgE and poor self-rated health was not attenuated by including corticosteroid dose or treatment with leukotriene-receptor antagonist (LTRA) in the analysis (coefficients changed by <5%, data not shown).

**Table 2:** Fixed effect coefficients (*b*) and 95% confidence intervals (bootstrapped-based *p*-values) for the association between self-rated health, sickness behaviour and inflammatory markers

	Time-pts	Obs	*b*	CI	*b* interaction variable × sex^a^	CI
Sickness behaviour	3	499	−0.46***	0.34; 0.58	−0.04	−0.21; 0.12
Energy	3	498	−0.26***	−0.33; −0.19	−0.04	−0.14; 0.06
Sleep	3	498	−0.18***	−0.24; −0.11	−0.02	−0.12; 0.07
Memory	3	496	−0.14**	−0.24; −0.04	−0.07	−0.20; 0.06
Fitness	3	497	−0.21***	−0.28; −0.13	0.02	−0.10; 0.13
Appetite	3	496	−0.24***	−0.34; −0.14	0.07	−0.06; 0.21
F_E_NO	5	821	0.05	−0.02; 0.12	−0.02	−0.12; 0.08
Perennial IgE^b^	2	309	−0.08	−0.26; 0.11	0.10	−0.10; 0.30
Seasonal IgE^b^	2	309	0.07*	0.00; 0.15	−0.07	−0.23; 0.10
Food IgE^b^	2	309	0.18*	0.01; 0.35	−0.18	−0.36; 0.01
Total IgE^b^	2	327	0.15	−0.06; 0.37	−0.07	−0.34; 0.19
S-ECP^b^	2	309	−0.09	−0.20; 0.01	0.07	−0.10; 0.24
P-EDN^b^	2	309	−0.05	−0.17; 0.07	−0.14	−0.50; 0.22

*Time-pts* number of time points included in analysis, *obs* number of observations in analysis, *CI* confidence intervals, *F*_*E*_ *NO* exhaled fraction of nitric oxide, *S-ECP* serum eosinophil cationic protein, *P-EDN* plasma eosinophil-derived neurotoxin

*Note*: Fixed effect coefficients (*b*) and 95% confidence intervals (bootstrapped-based *p*-values) for the association between self-rated health and composite score of sickness behaviour, the individual items included in sickness behaviour separately, F_E_NO,IgE, S-ECP and P-EDN. All analyses were adjusted for BMI and age

^a^ Interaction term between independent variable and sex (woman = 1)

^b^ All IgE variables, S-ECP and P-EDN values were z-transformed to facilitate interpretation of *b*-coefficients

*p < 0.05; **p < 0.01; ***p < 0.001

Poor self-rated health was significantly associated with higher values for the sickness behaviour composite variable in both men and women (Table 2). When analyzed separately, all individual items in the composite sickness variable were significantly associated with self-rated health.

No significant associations between the composite sickness behaviour variable and the inflammatory markers were found (Table 3). Individual sickness behaviour items were associated with some inflammatory markers, especially in women. Specifically, a high F_E_NO was associated with poor fitness (*b*: −0.13, 95% CI −0.26; −0.01, *p* = 0.036) and low appetite (*b*: −0.13, 95% CI −0.26; −0.01, *p* = 0.039) in women. Similarly, perennial IgE was associated with low energy (*b*: −0.33, 95% CI −0.65; −0.01, *p* = 0.045) in women. Higher levels of P-EDN was associated with low energy in both men and women (*b*: 0.22, 95% CI 0.07; 0.39, *p* = 0.005).

**Table 3:** Fixed effect coefficients (*b*) and 95% confidence intervals (bootstrapped-based *p*-values) for the association between sickness behaviour and inflammatory markers

	Time-pts	Obs	*b*	CI	*b* women^a^	CI
F_E_NO^b^	5	494	0.04	−0.02; 0.09	−0.07	−0.17; 0.03
Perennial IgE^b^	2	308	0.13	−0.08; 0.34	−0.21	−0.45; 0.03
Seasonal IgE^b^	2	308	−0.06	−0.15; 0.03	−0.16	−0.34; 0.01
Food IgE^b^	2	308	−0.07	−0.35; 0.21	−0.03	−0.35; 0.28
Total IgE^b^	2	326	−0.15	−0.06; 0.36	−0.07	−0.34; 0.19
S-ECP	2	308	0.15	−0.01; 0.31	−0.13	−0.35; 0.10
P-EDN	2	308	−0.05	−0.17; 0.07	−0.12	−0.71; 0.48

*BMI* body mass index, *Time-pts* number of time points included in analysis, *obs* number of observations included in analysis, *CI* confidence intervals, *F*_*E*_ *NO* exhaled fraction of nitric oxide, *S-ECP* serum eosinophil cationic protein, *P-EDN* plasma eosinophil-derived neurotoxin

*Note*: Fixed effect coefficients (*b*) and 95% confidence intervals for the association between sickness behaviour vs. F_E_NO, IgE, S-ECP and P-EDN. All analyses were adjusted for BMI and age

^a^ Interaction term between independent variable and sex (woman = 1)

^b^ All IgE, S-ECP and P-EDN values were z-transformed to facilitate interpretation of regression coefficients

### Association between change in self-rated health and change in inflammatory factors and sickness behaviour

An increase in perennial IgE was significantly correlated with a worsening of self-rated health (*ρ*: 0.16, *p* = 0.04). Likewise, an increase in the sickness behaviour composite variable was significantly associated with a worsened self-rated health (*ρ*: 0.21, *p* = 0.01) as well as to a decrease in energy (*ρ*: −0.19, *p* = 0.04). There were no other significant associations between change in self-rated health and change in other inflammatory markers or sickness behaviour, including the individual sickness items sleep, memory, fitness or appetite. Associations are presented in Table 4.

**Table 4:** Correlations between change in sickness behaviour, inflammatory markers and change in self-rated health between baseline and follow-up

	*ρ*	*p*-value
Sickness behaviour	0.21	0.007*
F_E_NO	0.07	0.39
Perennial IgE	0.16	0.04*
Seasonal IgE	−0.11	0.19
Food IgE	−0.01	0.87
Total IgE	0.08	0.31
S-ECP	0.14	0.09
P-EDN	−0.04	0.65

*F*_*E*_ *NO* exhaled fraction of nitric oxide, *S-ECP* serum eosinophil cationic protein, *P-EDN* plasma eosinophil-derived neurotoxin

*p < 0.05; **p < 0.01; ***p < 0.001

## Discussion

In this 12-month longitudinal study, poor self-rated health was associated with more pronounced sickness behaviour, especially lower ratings of energy levels, and a worsening of sickness behaviour was associated with a worsening of self-rated health over time. In addition, poor self-rated health was associated with high levels of seasonal IgE and food IgE in both men and women and increased levels of perennial IgE was associated with a worsening of self-rated health over time. However, the data did not support the hypothesis that higher levels of the inflammatory markers F_E_NO, S-ECP and P-EDN would be associated with poor self-rated health. Thus, here we suggest the importance of sickness behaviour as a determinant of self-rated health by showing that the variables co-vary over a 1-year period in patients with allergic asthma. Furthermore, this study reveals a stronger association between IgE antibodies and self-rated health, compared to variation in the inflammatory markers F_E_NO, S-ECP or P-EDN, even though the importance of specific IgE for perceived health needs further investigation.

One possible explanation as to why no association between the inflammatory markers F_E_NO, S-ECP and P-EDN was found is that the patients have adapted in their behavioural and subjective response to the chronic allergic inflammatory signal, if this reaches the brain similar to what is known for pro-inflammatory cytokines.^17^ In fact, the majority of patients rated their health as “quite good” already at the starting point of the study in spite of the asthma diagnosis. Other studies have shown that when patients with cancer had time to adapt to their disease they reported their health-related quality of life as good as before diagnosis despite physical limitations and adaptation; a phenomenon referred to as “response shift”.^24,25^ Similarly, fatigue and lack of energy in patients with cancer have been shown to be subject to recalibration, so that the patients’ prechemotherapy symptoms were rated milder when viewed in retrospect.^26^ Thus, inflammatory activity, as reflected by levels of F_E_NO, S-ECP and P-EDN, may have relatively less impact on subjective health parameters in a chronic condition such as asthma where response shifts in subjective parameters occur. Another possible explanation to the lack of association between the inflammatory markers and self-rated health in the present study is their temporal stability so that inflammatory markers that fluctuates more rapidly than what is relevant for self-rated health and sickness behaviour would demonstrate a lower association with these outcomes in these patients with relatively stable disease. It could also be explained by differences in their influence on factors determining self-rated health. In the present study no associations between IgE or any of the inflammatory markers and sickness behaviour were found. However, in an earlier report from the same study sample, a reduction in perennial, total and all specific IgE was significantly associated with an improvement in asthma-related quality of life,^27^ suggesting that certain IgE measures might be related to other factors than sickness behaviour relevant for subjective health perception. Hence, measuring levels of IgE could be a valuable clinical complement when evaluating variables associated with asthma-related quality of life, self-rated health and other patient-reported outcomes in asthma^27^ and future research might delineate if IgE differs from other disease-specific variables in its relation to indices of subjective health.

In this study, asthma control, which is a clinical relevant measure, was not included. In a previous study, a decrease in IgE concentrations was found to correlate with an improvement on both asthma control and asthma-related quality of life.^27^ Furthermore, poor asthma-related quality of life has been shown to be associated with both poor self-rated health and increased sickness behaviour.^28^ Thus, it could be of interest to investigate the relationship between asthma control and patient-reported outcome measurements in future studies.

In contrast to the inflammatory markers S-ECP and P-EDN, which reflect systemic inflammation, F_E_NO measures local inflammatory processes in the airways.^29^ In spite of its organ specificity, F_E_NO was not associated with self-rated health or sickness behaviour in the present study. This partly contrasts studies that have shown a link between a higher F_E_NO and negative affect, anxiety and acute stress,^30–32^ although another study found no effects of examination stress on F_E_NO in asthmatic student, whereas a small reduction was observed in non-atopic subjects.^33^ Also, although F_E_NO was associated with asthma symptom control and IgE levels, no significant association was found between F_E_NO and asthma-related quality of life in the earlier report from the study.^27^ Therefore, patient-reported outcomes and FENO might be considered to be used as complementary measures since they assess different dimensions of asthma in addition to the traditional measures.

Higher sickness behaviour was strongly associated with poorer self-rated health in the present study as hypothesized consonant with an earlier observation that self-rated health was associated with a similar composite variable of sickness behaviour in a cross-sectional study of primary care patients.^20^ The results are consistent with a causal relationship between the two, as an increase in sickness behaviour was significantly associated with a decrease in self-rated health over time. This is new and important information given the need to understand determinants of health and other patient-reported outcomes and as previous reports of the relation between sickness behaviour and self-rated health have been based on cross-sectional data. Also the relation between self-rated health and inflammation has mainly been investigated in cross-sectional studies. A recent longitudinal study demonstrated a stable relationship between poor self-rated health and higher IL-6 in older adults over several measurements^34^ but this study did not include any measures of sickness behaviour. Because previous studies show that pro-inflammatory cytokines both causes sickness behaviour and poorer self-rated health,^35,36^ the corresponding relationships should be investigated in allergic asthma. Notably, there is some support that asthma is related to increases also in systemic pro-inflammatory cytokines such as IL-1 and TNF-alpha^37^ known to induce sickness behaviour.

This study has several strengths. First, this is the first longitudinal, multicentre study investigating the relationship between self-rated health, sickness behaviour and disease-specific inflammatory markers in primary care patients with allergic asthma with all paired serum measurements analyzed side by side. Second, the patient sample was, by and large, representative for patients with asthma treated in primary health care in Sweden.^38^ Information of anti-inflammatory treatment was registered and the association between high levels of IgE and poor self-rated health remained significant also when adjusted for LTRA and corticosteroid treatment. However, as patients with severe asthma were excluded, the present findings should be viewed in light of the fact that the study population was a rather homogenous group of patients with well-managed mild to moderate allergic asthma with inhaled corticosteroids (ICS) and LTRA as anti-inflammatory treatment. This might be a limitation because severe asthma has been associated with increased blood eosinophil count, high S-ECP and P-EDN, as well as increased levels of IgE and F_E_NO compared to patients with mild to moderate asthma.^39,40^ Including also patients with a more prominent inflammation and more pronounced sickness behaviour would increase the variation in the investigated variables and would have increased the power in the analyses. Another limitation may be that negative affect was not included in the composite variable of sickness behaviour used in the present study. In recent years there has been a growing amount of evidence that both negative and positive affect are an important part of sickness behaviour.^9,21,41^ A more comprehensive measure of sickness behaviour including measures of affect, like the Sickness Questionnaire,^36^ could therefore be useful in order to delineate behavioural factors that co-vary with self-rated health and inflammation in patients with asthma. In future studies, this validated instrument could provide a more accurate measure of sickness behaviour instead of using a composite variable.

In conclusion, this study corroborates the importance of sickness behaviour as a determinant of self-rated health by showing that the variables co-vary over a 1-year period in patients with allergic asthma. The importance of specific IgE for perceived health in primary care patients with mild to moderate asthma needs further investigation.

## Methods

### Participants

The NOAK study (optimization of anti-inflammatory asthma treatment using exhaled nitric oxide to improve asthma-related quality of life within primary health care) was a randomized, controlled trial on F_E_NO-guided treatment with asthma-related quality of life and asthma symptom control as endpoints. Details on sampling procedure and data collection have been described elsewhere.^38^ A brief description of methodological procedures is given here. Informed written consent was obtained prior to the start of the study from all participants. The study was registered in clinical trials, NCT00421018, and was approved by the regional ethics committee in Stockholm (Dnr: 2006/185–31).

A sample of 181 patients with asthma (87 women, 94 men) aged 18–64 years were recruited at 17 primary health care centres in seven different county councils in central and southern Sweden from November 2006 to March 2010. All participants had a diagnosis of asthma and had confirmed IgE sensitization to at least one airborne perennial allergen. The participants were non-smokers since at least 1 year before inclusion. Included patients had a previous smoking history of maximum 10 pack-years.

### Procedure

All participants were on regular treatment with ICS which had been prescribed for at least 6 months. Participants being treated with combination inhalers (corticosteroids plus long-acting beta-2-agonists) had to withdraw the long-acting beta-2-agonist component and switch to corresponding single corticosteroid inhaler. The participants were randomized into two groups by lottery in a straight randomization. The groups differed in how the anti-inflammatory treatment, dose of ICS and LTRA (montelukast 10 mg daily), was guided. In the F_E_NO-guided treatment group (*n* = 93) the anti-inflammatory treatment was adjusted on the basis of F_E_NO, whereas treatment in the control group (*n* = 88) was adjusted based on symptoms according to routine clinical practice.^38^ Methods were performed in accordance with relevant regulations and guidelines. As there were no significant effects of treatment guidance on self-rated health, sickness behaviour nor any of the IgE-variables, S-ECP or P-EDN, groups were combined in all analyses in this present report and treatment is included as a confounder.

The study included in total six examinations at the health care centre for the participants: inclusion, visit 1 (baseline), visit 2 (2 months), visit 3 (4 months), visit 4 (8 months) and visit 5 (12 months).

### Measurements

#### Self-rated health

Subjective health was measured at all visits using the question “How would you rate your general state of health?”. The response alternatives were: very good (coded as 1), rather good (2), neither good nor poor (3), quite poor (4) and poor (5).

#### Sickness behaviour

A composite measure similar to that used in Undén et al.^20^ was used to assess sickness behaviour. The composite measure included weighted means of the answers to the questions “How satisfied are you with your situation regarding the following aspects: energy/sleep/fitness/appetite and memory”.^20^ The responses on each item were rated on a Likert scale ranging from “excellent, could not be better” (1) to “very poor” (7). Sickness behaviour was assessed at visit 1, 3 and 5.

#### IgE, S-ECP and P-EDN

Venous blood for analysis of levels of IgE, S-ECP and P-EDN was sampled in EDTA-containing and serum gel-containing tubes, respectively. The samples were centrifuged and initially stored at −20 °C before aliquots were transferred to −70 °C until analysis. ImmunoCAP Phadiatop® (dog, cat, horse, birch, timothy, *Dermatophagoides pteronyssinus*, *Dermatophagoides farina*, *Cladosporidium herbarum*, mugworth), ImmunoCAP fx5® (cow’s milk protein, egg white, peanut, soy, wheat, fish), total IgE, s-ECP and p-EDN were analyzed from the blood samples drawn at visit 1 (baseline) and 5 (12 months). Specific IgE antibodies were grouped into three different categories: *perennial* (cat, dog, horse, mite × 2, cladosporium), *seasonal* (birch, timothy, mugworth) and *food* (cow’s milk protein, egg white, peanut, soy, wheat, fish).

Serum samples for IgE and ECP were analyzed in a Phadia 100 system with ImmunoCAP reagents (Immunodiagnostics, Thermo Fischer Scientific, Uppsala, Sweden). Plasma samples for EDN were analyzed in a sandwich-ELISA (Diagnostics Development, Uppsala, Sweden). Each patient’s samples, from baseline and 12 months, were analyzed side by side in the instrument for all measurements.

#### Fraction of exhaled nitric oxide

F_E_NO was measured at all visits (NIOX MINO: Aerocrine AB, Solna, Sweden). Participants were asked to inhale to total lung capacity through the NIOX MINO and then exhale for 10 s at 50 ml/s (assisted by visual and auditory cues). F_E_NO (parts per billion) was recorded as mean value from two (alternatively three if more than 5% variation was seen between the two samples) successive measurements. F_E_NO values were standardized according to predicted normal values in non-atopic adult individuals according to height and age.^42^ F_E_NO was measured in all participants but the result was blinded for clinicians and patients in the control group.

#### Background factors

Age was retrieved from each participant’s personal identity number. Height and weight were measured at the first visit and used to calculate body mass index (kg/m^2^). Educational level was retrieved from questionnaires and classified into three levels, from compulsory school (1) to university (3).

### Statistics

To test if self-rated health, sickness behaviour or any of the inflammatory markers changed during the course of the study, mixed effect regression analyses was used including time as an independent dummy variable.

The overall associations between inflammatory markers, sickness behaviour and self-rated health were calculated using mixed effect regression models. Each inflammatory marker was analyzed separately as independent variables and the models included patient ID, sex and an interaction term between sex and the independent variable to test if the associations differed between men and women. In addition to the composite score of sickness behaviour, the individual items included in the score were investigated as dependent variables in explorative analyses. Corticosteroid dose and treatment with LTRA was included in a follow-up analysis to investigate if anti-inflammatory treatment influenced association between inflammatory factors and self-rated health. All models were adjusted for age and BMI. Due to the non-normal properties of the included variables, the *p*-values in all mixed effect regression models were estimated by bootstrap with 1000 repetitions.^43^ Crude IgE, s-ECP and p-EDN values were z-transformed prior to analysis to facilitate interpretation of regression coefficients (*b*).

To investigate if changes in inflammatory factors were associated with a change in sickness behaviour and self-rated health, the difference in inflammatory factors, sickness behaviour and self-rated health between visit 1 and visit 5 was calculated and the delta values were correlated. Due to the non-normal properties of the delta values, Spearman rank correlations were used. STATA® 14.0 (StataCorp, LP, TX, USA) were used for all analyses. An α-level of 0.05 was used to test for significance.

### Data availability

All relevant data are available from the corresponding author on request.

## Electronic supplementary material

Study protocol
