# Supplementary material for: Associations between self-rated health, sickness behaviour and inflammatory markers in primary care patients with allergic asthma: a longitudinal study
Source: NPJ Prim Care Respir Med. 2017 Dec 18;27:67. doi: 10.1038/s41533-017-0068-0 (PMC5735192; doi:10.1038/s41533-017-0068-0)
Supplement: Supplementary file 1 — Study protocol [file 41533_2017_68_MOESM1_ESM.doc]

**Study protocol** 070923

**Title:**

**Optimization of Asthma Treatment Through Exhaled NO for Increased Asthma-Related Quality of Life (NOAK)**

**Responsible:**

Kjell Alving, Professor

Department of Physiology and Pharmacology

Karolinska Institutet, 171 77 Stockholm

Phone. 08-524 879 51

Fax 08-33 22 78

E-mail kjell.alving@ki.se

Jörgen Syk, MD, PhD student

Runby health center

Box 474, 194 04 Upplands Väsby

Visiting address: Runby square 9

Phone. 08-590 990 60

Fax 08-590 917 65

Email jorgen.syk@ptj.se

Anna-Lena Undén, Research Director, PhD

Center for General Medicine

Alfred Nobels Allé 12

141 83 Huddinge

Phone. 08-524 887 57

Fax 08-524 887 60

Email anna-lena.undén@ki.se

Gunnar Johansson, PhD

Nyby Health Center, Heidenstamsgatan 69

754 27 Uppsala

Phone. 018-611 75 13

Email gunnar.johansson@pubcare.uu.se

Partners:

Mats Lekander, Section Manager, Department of Clinical Neuroscience, Karolinska Institutet

Juha Kere, Professor, Department of Biosciences and Nutrition, Karolinska Institutet

Marianne Heibert Arnlind, PhD student, Center for Allergy Research, Karolinska Institutet

Aerocrine AB, Solna

Phadia AB, Uppsala

Meda AB, Solna

Participating primary health care centers:

Runby primary health care Center, Upplands Väsby

Kista primary health care Center, Kista

The doctors in Österåker, Åkersberga

Bålsta primary health care Center, Bålsta

Additional health care centers will be recruited

**Background:**

Untreated patients with allergic asthma develop an eosinophilic inflammation of the respiratory tract and display elevated amounts of nitric oxide (NO) [1]. Treatment with anti-inflammatory drugs (steroids and anti-leukotrienes) lowers levels of NO, while other types of asthma medications do not affect the NO value. According to current guidelines for asthma treatment, anti-inflammatory treatment is primarily controlled by means of symptom, as well as based on results from pulmonary function tests and PEF measurements. However, in many patients, an airway inflammation appears to be present, still established guidelines have been followed, risking future permanent changes in the respiratory tract and suboptimal asthma control [2]. Exhaled nitric oxide (NO) has been shown to reflect the eosinophilic inflammation in the respiratory tract [3], and there are now easy-to-read measuring equipment and with good reproducibility that can be used at a primary health care center. Two recently published studies have shown improved treatment of asthmatics by controlling anti-inflammatory treatment based on the value of exhaled NO instead of the traditional way, where treatment is predominantly guided based on symptomatology [4, 5].

Here, we want to study the clinical benefit of controlling steroid treatment in a primary health care setting in patients with allergic asthma using exhaled NO, and within the scope of this relatively large study, we also want to include some other important issues relevant to these patients.

An individual's appraisal of his own global health (self-rated health) has been shown to predict future morbidity and mortality. In many cases, the predicitve value of self-rated health equals or surpasses objective values and medical examinations. However, the biological background of this phenomenon is largely unknown. Today there is enough data to suggest that immunomodulation caused by psychosocial stress can lead to changes in health. In one of our recent studies, lower self-rated health was found to be associated with higher circulating levels of the pro-inflammatory cytokines TNFα and IL-1β, but the relationship was only significant in women [6]. Pro-inflammatory cytokines can be stimulated by negative emotions, stressful experiences, chronic inflammation as well as infections, and many allergic patients as well as the clinicians seem to experience a connection between allergy and stress. It is quite possible that allergic disease in itself contributes to increased stress because it has been shown that proinflammatory cytokines activate the so-called HPA (hypothalamus, pituitary, adrenal) system. Certain cytokines appear to be particularly important in an atopic response, including IL-4 and IL-5. Allergic patients, however, may have elevated levels of general proinflammatory cytokines, such as IL-1β and TNFα [7, 8]. This is interesting from several perspectives. First, it is well known that these cytokines affect brain structures that are important for the regulation of activity level, sleep and mood, and that they activate the HPA system. Secondly, it has often been argued that stress can affect allergic symptoms or underlying inflammation, which later studies confirm [9, 10]. In many cases, however, the causal link between stress and allergy remain unclear. Therefore, there are important theoretical reasons to study the connection between immune activity, allergic symptoms and subjective variables such as self-rated health and perceived quality of life. The SBU's Summary on Treatment of Asthma and COPD from 2000 highlights the importance of using self-rated health and different patient reported outcomes such as different measurements of quality of life in order to study the effect of different treatment programs. This research can now potentially be strengthened further since we now have access not only to systemic inflammatory markers but also to a local marker of airway inflammation, exhaled NO. Another factor that appears to contribute to the development of asthma is obesity [11], and obesity is also associated with increased levels of circulating cytokines, such as TNFα and IL-6 [12]. Thus, when studying the relationship between stress, circulating cytokines and asthma symptoms, body mass index (BMI) will also need to be examined.

Although asthma and allergy are more common among boys than girls both before and during puberty, it seems that receiving an asthma diagnosis in adulthood is more common among women. Of importance is also the fact that important that asthma severity improves in men, but deteriorates in women, from childhood to adulthood [13]. Since we have seen a strong link between self-rated health and circulating cytokines in women, but not in men, these psychoneuroimmunological factors could be a possible explanation for the poorer asthma development in women. Although asthma has long been defined as a chronic inflammatory disease, many asthma patients today are treated with relatively expensive so-called combined medication, where the inhaler contains both a steroid and a long-acting β2 stimulator (LABA). There is a clear risk that a continuous inflammation will not be noticeable because of the bronchodilator component of the combination inhaler, with potential risks later on. In addition, it has been shown that LABA may have proinflammatory effects [14], especially in a fraction of the population (about 16%) with a certain genotype of the β2 receptor [15]. It could therfore be of use to primarily control the bronchial inflammation in order to minimize the use of β2 stimulators in general. The search for risk genes for asthma development has recently shown other interesting gene candidates, such as GPRA [16]. Certain haplotypes of the GPRA gene have been shown to be associated with both higher levels of IgE and asthma. It is important to evaluate both medical outcomes and economical outcomes. Asthma causes financial consequences for both the patient, the primary care and society at large and it is important that these outcomes can be described. To the best of our knowledge, no study has been made of the economic consequences of controlling steroid use in asthmatics by using exhaled NO in relation to controlling the treatment in a traditional manner.

**Aims:**

*Primary aim:*

– Can control of anti-inflammatory treatment using exhaled NO lead to improved asthma-related quality of life in patients with allergic asthma?

*Secondary aims:*

– Will the number of asthma exacerbations and / or total steroid consumption decrease in the group in which treatment is controlled according to the value of the exhaled NO (NO group)?

– Will the consumption of bronchodilator drugs be less in the NO group?

– Will asthma symptom control and asthma-related quality of life (measured by questionnaires) be better in the NO group?

- Will lung function improve in the NO group?

- Will self-rated health improve and circulating cytokines decrease in the NO group?

-Will levels of IgE antibodies decrease in the NO group?

- Can the value of NO be normalized in the NO group? Unless, why not?.

- Are there any associations between self-rated health, stress, obesity and various inflammatory markers (NO, CRP and cytokines)?

- Will the cost for drugs, healthcare and sick leave be reduced in the NO group?

**Inclusion criteria:**

**-**Age 18-64

**-**Clinical diagnosis of asthma

**-**Glucocorticoid treatment > 6 months.

**-**Verified Allergy (tested with Immuno CAP™ Rapid, Wheeze/Rhinitis Child)

-Read and speak Swedish

-Written consent before the start of the study

**Exclusion criteria:**

-Smoking participants (not allowed to have smoked regularly during the past year and no more than ten package years in total)

-Current regular treatment with long-acting beta2-agonist

-Treatment with Singulair only or in combination with glucocorticoids

-Taking part in other research study

-Pregnancy or breast-feeding

-Unstable Asthma (≥4 cures with prednisolon last year or hospital care during the last 6 months)

**Randomization:**

Patients are randomized at each primary health care center, by lottery using sealed envelopes which are assigned to the primary care health center, 50/50 distribution between the two study arms

**Study design:**

Approximately 200 patients will be included at 12-15 different health centers. After an initial preparatory visit, participants will be monitored for 5 visits during 12 months (0.2.4.8 and 12 months). Patients should have received an asthma diagnosis by a physician and be on intermittent or continuous steroid therapy for at least six months, have a verified allergy to at least one airborne perennial allergen, be between 18-64 years, be non-smoking for at least one year and in total not have been smoking for more than a total of 10 years during the lifetime. All patients treated with so-called combination medication (steroid and long-acting β2 stimulator in the same inhalator) should switch steroids and β2 stimulants in separate inhalers within four weeks prior to visit 1. If this is not clinically possible, the patient cannot participate in the study.

Upon inclusion, the participants will continue treatment with the same steroid dosage as they had before initiation of the study. Patients on Becotide should switch to Pulmicort Turbuhaler in equivalent dose. Patients who use Ventoline Diskus 0.2 mg / dose may continue with it, while other patients are given a free of charge Buventol easyhaler® (salbutamol 0.2 mg / dose). The inhaler should accompany the patient to each return visit so that the counter on the inhalator can be read by the study leader. Old already used inhalers must also be brought back to the study leaders. It is important to encourage the participants to use β2-stimulant when they have any asthma symptoms.

There will be two study arms; one where the value of exhaled NO is blinded for both the patient and the doctor, and one where the NO values are reported openly. In the group with the blinded NO value, asthma treatment is controlled traditionally by means of symptoms, possibly PEF measurement and spirometry. The dosage steps must be followed but there is no limitation on how to "jump" between the doses. In the second group, the NO values are used to control the steroid dose.

At a NO value ≥ 26 ppb (men) and ≥ 24 ppb (women), treatment will be increased by one dose step and when the NO value is <21 ppb for men and <19 ppb for women, a reduction with one dose step will be made. At intermediate NO values, the patient will remain on the same dose stage.

If patients have clearly elevated levels of NO, ≥ 32 ppb for men and ≥ 30 ppb for women, and are at dose level 1, treatment will be increased by two doses, otherwise all changes (up or down) will be to the adjacent dosage steps. Patients with an elevated NO value which are being treated at doses 4 or 5 and on average do not use more than two inhalations/week with β2 stimulators, will not be able to increase to the next dose stage but stop at the current dosage regimen.

Patients in whom steroid treatment and anti-leukotriene alone is not enough to control the symptoms satisfactorily (and therefore may require addition of long-acting β2 stimulators) should discontinue the study, including those unable to use Singulair due to side effects.

| Dosage steps (µg/daily) | 1 | 2 | 3 | 4 | 5 | 6 |
| --- | --- | --- | --- | --- | --- | --- |
| Pulmicort Turbuhaler Gina Easyhaler | 0 | 200 | 400 | 800 | 800 +  Singulair | 1600 +  Singulair |
| Flutide Diskus | 0 | 100 | 250 | 500 | 500 +  Singulair | 1000 +  Singulair |
| Asmanex | 0 | 100 | 200 | 400 | 400 + Singulair | 800 + Singulair |

Patients who suffers from a significant respiratory tract infection should cancel the visit until they feel healthy again to avoid the NO measurement being affected too much (respiratory tract infections can result in increased NO values). However, the shift in time frame should not exceed four weeks.

Rescheduled visits cannot be moved further than two weeks before the original planned date for the visit. Patients with birch pollen allergy should not commence the study if the first two months (the initiation phase) coincide with the birch pollen season and, if possible, avoid follow-up visits during this season as well.

*Exacerbations:*

In case of asthma deterioration, the patient should firstly contact the doctor who is responsible for their treatment during the study. This will in most cases be the patient's regular GP. If increasing the inhalation steroid is sufficient, this should be done for 14 days with possibility to extension if necessary (in consultation with his doctor). If oral steroid treatment is prescribed, the inhalation steroid should be kept unchanged. Treatment with long-acting bronchodilator medication must not be initiated. Patients will receive a card with the above instructions that should be shown upon contact with the healthcare provider in case of asthma impairment.

The participants’ are obliged to always inform the study doctor if the dosage of steroids has changed for some reason and the information must be recorded in the logbook.

In case of other health issues, the patient should primarily attend his regular GP (during office hours) or available local health centers or hospitals during nighttime.

Any kind of treatment with cortisone should be noted in the study form.

*Allergic rhinitis och conjunctivitis*

Antihistamines, local steroids and sodium chromoglycate may be used for the treatment of rhinitis and conjunctivitis as usual, but treatment with cortisone injections or singulair should not be used. If the symptoms are pronounced, cortisone can be given briefly in tablet form and this should of course be noted in the study form.

*Adverse drug reactions*

Possible side effects due to elevated doses of cortisone or addition of Singulair are noted in a special form. All serious adverse drug reactions should be reported to the Swedish Medicines Agency (see FASS). Serious adverse drug reactions which are possibly, probably or definitively related to Singulair®, must be reported in writing to MSD within 24 hours after they come to notice (tel. 08-626 14 00, e-mail: [info_sverige@merck.com](mailto:info_sverige@merck.com)). (Serious adverse drug reactions in human studies include the following: if the reaction is fatal, life threatening, invalidating, causing malformations, if hospitalization or prolonged hospitalization is needed or if the reaction results in intoxication or causes cancer.)

If the study participants interrupt or are excluded from the study, date and cause must be entered in special forms.

**Procedure**

Initially, a preparatory visit, **visit 0**, will be given where the participant will receive oral and written information about the study, and to find out about any obstacles for participation. Allergy test is done with Immuno CAP ™ Rapid, Wheeze / Rhinitis Child on capillary blood to confirm the presence of allergy. Randomization by lottery is performed allocating the participants to one of the study groups and the participants are given a card with study information and information about the treatment that can be given within the frame of the study in case of asthma deterioration. The card is to be shown at any contact with other health care centers in addition to the study doctor. Participants on a so-called combined medication (Symbicort or Seretide) must switch to separate cortisone and short-acting bronchodilator-inhalers (Buventol Easyhaler) and are required to have used this medication for at least four weeks before visit 1. All data should be entered in a detailed study form where data regarding body weight, length, etc. are noted as well.

**Visit 1** is scheduled 2-4 weeks after the preparatory visit. It is important that the participant has been free from infection (upper and lower respiratory tract infection) during the last two weeks before visit 1. If they have been treated with any antibiotics, the treatment must be completed no later than four weeks before the visit. Notes will be taken regarding drug consumption, including consumption of β2-stimulatory medication, contact with health care if applicable and sickness absence. Asthma classification and measurement of exhaled NO will be performed.

Data from the NO measurements is stored on two so-called Smart cards that are stuck in the NO meter before measurement, one for open measurements (the value is also shown on the display) and one for blind measurements (the value is not shown on the display). If NO is measured outside the study, this should be done without registration on the smart card. Spirometry will be performed. Vital capacity with maximum slow exhalation will be calculated from the spirometry values, forced exhalation and a reversibility test will be performed during the spirometry. Bronchodilators (inhalation of terbutaline 0.5 mg x 2 or salbutamol 0.4 mg x 2) will be used during the test and there will be a 15 minutes interval between the inhalation and the repeated measurement. Paper copies of the spirometry results are stored in the patient file. Importantly, the same regime with bronchodilation will be used for visit 1 and 5. Hedenström’s reference values are used as a reference. The questionnaires on asthma-related quality of life (Juniper mini-AQLQ), overall quality of life, self-rated health, stress, sleep quality, coping ability, compliance to medication; asthma control (Juniper ACQ), health economics (EQ-5D) and allergy exposure should be completed.

A logbook is distributed where the patient should record any medical contacts, changes in drug therapy and sick leave. The logbook will be used in between study visits. In the “open” NO group, dosage of cortisone is adjusted based on the NO value measured. In the group with hidden NO, the cortisone dose is adjusted if needed, in the same manner as in traditional asthma monitoring according to regular practice. The participant will be given paper copies of the referrals needed for venous blood sampling at the local laboratory.

At **visits 2**, two months later, questionnaires about asthma control (ACQ) and allergy exposure questionnaires will be completed. Drug use, including consumption of β2-stimulants and possible contact with healthcare and any sickness absence is noted. In the study group with the “open” NO-value, NO measurement is performed and the cortisol dose is adjusted based on the measured NO value. In the group with “hidden” NO value, the cortisone dose is adjusted if needed, in the same manner as used in traditional asthma monitoring according to regular practice.

Subsequent visits are made after 4, 8 and 12 months. The visit at 4 months (**visit 3**) has the same content as visit 1, except for spirometry and sampling. The eight-month visit (**Visit 4**) has the same content as Visit 2, and the final 12-month visit (**Visit 5**) has the same content as at Visit 1.

Doctors will perform the visit 0.1 and 5 and preferably other visits but visit 2.3 and 4 may, if needed, be administered by an asthma nurse in consultation with the doctor in charge.

Venous blood samples from the participants will be sampled at the laboratory on two occasions, 0 and 12 months. The blood sample will be taken in the morning and then transported to the KI biobank for freezing and later analysis. Analysis will be performed with Immuno CAP ™ Phadiatop (Phadia), to determine the type and the degree of allergy. High sensitivity CRP and different cytokines (TNFα, IL-1β, IL-6, IFN-γ, IL-2, and IL-5) are analyzed in order to measure levels of inflammation. Haplotype analysis of the GPRA gene and genotyping of the β2 receptor will also be performed.

|  | **Visit 0**  **Inclusion** | **Visit 1**  **Baseline** | **Visit 2**  **2 months** | **Visit 3**  **4 months** | **Visit 4**  **8 months** | **Visit 5**  **12 months** |
| --- | --- | --- | --- | --- | --- | --- |
| **Allergy test,**  **capillary sample** | X |  |  |  |  |  |
| **Questionnaire** | X | X | X | X | X | X |
| **Spirometry** |  | X |  |  |  | X |
| **NO measurement** |  | X | X | X | X | X |
| **Questionnaire,**  **Quality of life** |  | X |  | X |  | X |
| **Questionnaire,**  **ACQ, exposure.** |  | X | X | X | X | X |
| **Venous**  **sampling** |  | X |  |  |  | X |

**Outcome measures**

*Primary*:

- Mini-AQLQ, differences in total score between groups and change over time within the group

*Secondary:*

- Number and severity of exacerbations, difference between groups.

- ACQ at 4.8 and 12 months, difference between groups and change over time within the group.

- Total use of β2 stimulants during the period 4-12 months, difference between the groups.

- Changes in lung function, difference between groups and change over time within the group.

- Cumulative consumption of steroids, difference between the groups.

- Changes in NO value, difference between groups and within each group.

-Change in IgE titers, difference between groups and change over time within each group.

- Compliance (in the questionnaire measured as doses not taken during last week before the visit).

- Self-rated health, difference between the groups and change over time within each group.

- Differences in drug costs, cost of healthcare and sick leave between the groups.

**Statistical analyses**

The material will be analyzed using different bivariate and multivariate analyses. Changes over time (baseline, 6 months, 12 months) with respect to different markers for asthma control, quality of life, stress, self-rated health and lung function for the two groups "NO-open" and "NO-blinded" will primarily be analyzed using paired t-test and chi-2 analysis. The difference between the groups regarding degree of change in the included variables will be analyzed using multiple variance analysis for repeated measurements. To study if any changes are dependent of gender, gender will be included as an explanatory variable.

The relationship between circulating cytokines, self-rated health, exhaled NO and different asthma markers will be analyzed in a first step by correlation analysis, first using the cross-sectional data and secondly using data measuring the changes in different variables over time and between groups. As knowledge is limited from previous asthma studies about how the variables are linked to each other, we will also develop models aimed at seeking to describe the "causal" relationships between different indicators of asthma control, exhaled NO, immunological markers, self-rated health and quality of life. The analyzes are based on canonical correlations (partial correlations). The advantage of working with this approach is that, apart from direct connection between different variables and the outcome variable, you can also get an idea of the indirect relationships that affect the current outcome variable (eg, degree of inflammation, exaggerated NO, self-rated health, quality of life) through other variables. In the analyzes, gender perspectives will be taken into account, which means that you do not only study differences between men and women, but also investigates whether the relationship and explanatory models differs or not when the model is adjusted for sex.

**References**

1. Alving, K., E. Weitzberg, and J.M. Lundberg, *Increased amount of nitric oxide in exhaled air of asthmatics.* Eur Respir J, 1993. **6**(9): p. 1368-70.

2. van den Toorn, L.M., et al., *Airway inflammation is present during clinical remission of atopic asthma.* Am J Respir Crit Care Med, 2001. **164**(11): p. 2107-13.

3. Mattes, J., et al., *NO in exhaled air is correlated with markers of eosinophilic airway inflammation in corticosteroid-dependent childhood asthma.* Eur Respir J, 1999. **13**(6): p. 1391-5.

4. Smith, A.D., et al., *Use of exhaled nitric oxide measurements to guide treatment in chronic asthma.* N Engl J Med, 2005. **352**(21): p. 2163-73.

5. Pijnenburg, M.W., et al., *Titrating steroids on exhaled nitric oxide in children with asthma: a randomized controlled trial.* Am J Respir Crit Care Med, 2005. **172**(7): p. 831-6.

6. Lekander, M., et al., *Self-rated health is related to levels of circulating cytokines.* Psychosom Med, 2004. **66**(4): p. 559-63.

7. De Amici, M., et al., *Variations in serum levels of interleukin (IL)-1beta, IL-2, IL-6, and tumor necrosis factor-alpha during specific immunotherapy.* Ann Allergy Asthma Immunol, 2001. **86**(3): p. 311-3.

8. Krouse, H.J., J.E. Davis, and J.H. Krouse, *Immune mediators in allergic rhinitis and sleep.* Otolaryngol Head Neck Surg, 2002. **126**(6): p. 607-13.

9. Lekander, M., *[The immune system is affected by psychological factors. High stress levels can change susceptibility to infection and allergy].* Lakartidningen, 1999. **96**(44): p. 4807-11.

10. Liu, L.Y., et al., *School examinations enhance airway inflammation to antigen challenge.* Am J Respir Crit Care Med, 2002. **165**(8): p. 1062-7.

11. Schaub, B. and E. von Mutius, *Obesity and asthma, what are the links?* Curr Opin Allergy Clin Immunol, 2005. **5**(2): p. 185-93.

12. Lee, Y.H. and R.E. Pratley, *The evolving role of inflammation in obesity and the metabolic syndrome.* Curr Diab Rep, 2005. **5**(1): p. 70-5.

13. Kjellman, B. and P.M. Gustafsson, *Asthma from childhood to adulthood: asthma severity, allergies, sensitization, living conditions, gender influence and social consequences.* Respir Med, 2000. **94**(5): p. 454-65.

14. Buchvald, F. and H. Bisgaard, *Comparisons of the complementary effect on exhaled nitric oxide of salmeterol vs montelukast in asthmatic children taking regular inhaled budesonide.* Ann Allergy Asthma Immunol, 2003. **91**(3): p. 309-13.

15. Israel, E., et al., *Use of regularly scheduled albuterol treatment in asthma: genotype-stratified, randomised, placebo-controlled cross-over trial.* Lancet, 2004. **364**(9444): p. 1505-12.

16. Laitinen, T., et al., *Characterization of a common susceptibility locus for asthma-related traits.* Science, 2004. **304**(5668): p. 300-4.
